# Report of the Largest Chinese Cohort With *SLC19A3* Gene Defect and Literature Review

**DOI:** 10.3389/fgene.2021.683255

**Published:** 2021-07-01

**Authors:** Jiaping Wang, Junling Wang, Xiaodi Han, Zhimei Liu, Yanli Ma, Guohong Chen, Haoya Zhang, Dan Sun, Ruifeng Xu, Yi Liu, Yuqin Zhang, Yongxin Wen, Xinhua Bao, Qian Chen, Fang Fang

**Affiliations:** ^1^Department of Neurology, National Center for Children’s Health, Beijing Children’s Hospital, Capital Medical University, Beijing, China; ^2^Department of Neurology, Children’s Hospital Affiliated to Zhengzhou University, Henan Children’s Hospital, Zhengzhou, China; ^3^Department of Neurology, Wuhan Children’s Hospital, Wuhan, China; ^4^Department of Neurology, Gansu Maternal and Children’s Hospital, Lanzhou, China; ^5^Jinan Pediatric Research Institute, Qilu Children’s Hospital of Shandong University, Jinan, China; ^6^Department of Neurology, Tianjin Children’s Hospital, Tianjin, China; ^7^Department of Pediatric Neurology, Peking University First Hospital, Beijing, China; ^8^Department of Neurology, Capital Institute of Pediatrics, Beijing, China

**Keywords:** *SLC19A3*, biotin-thiamine responsive basal ganglia disease, Chinese cohort, outcome predictors, literature review

## Abstract

Thiamine metabolism dysfunction syndrome 2 (THMD2) is a rare metabolic disorder caused by *SLC19A3* mutations, inherited in autosomal recessive pattern. As a treatable disease, early diagnosis and therapy with vitamin supplementation is important to improve the prognosis. So far, the reported cases were mainly from Saudi Arab regions, and presented with relatively simple clinical course because of the hot spot mutation (T422A). Rare Chinese cases were described until now. In this study, we investigated 18 Chinese THMD2 patients with variable phenotypes, and identified 23 novel *SLC19A3* mutations, which expanded the genetic and clinical spectrum of the disorder. Meanwhile, we reviewed all 146 reported patients from different countries. Approximately 2/3 of patients presented with classical BTBGD, while 1/3 of patients manifested as much earlier onset and poor prognosis, including infantile Leigh-like syndrome, infantile spasms, neonatal lactic acidosis and infantile BTBGD. Literature review showed that elevated lactate in blood and CSF, as well as abnormal OXPHOS activities of muscle or skin usually correlated with infantile phenotypes, which indicated poor outcome. Brainstem involvement on MRI was more common in deceased cases. Thiamine supplementation is indispensable in the treatment of THMD2, whereas combination of biotin and thiamine is not superior to thiamine alone. But biotin supplementation does work in some patients. Genotypic-phenotypic correlation remains unclear which needs further investigation, and biallelic truncated mutations usually led to more severe phenotype.

## Introduction

Thiamine transporter-2 deficiency (THMD2, OMIM#607483), also called biotin thiamine responsive basal ganglia disease (BTBGD) was first described by Ozand et al. (1998). The disease is characterized by acute/subacute or recurrent episodes of encephalopathy, often triggered by febrile illness, companied with dystonia or hypotonia, bulbar dysfunction, ataxia, and seizures. If not treated in time, encephalopathies lead to permanent dystonia and might cause coma and death in severe conditions. Zeng et al. (2005) first revealed that the *SLC19A3* gene is the causative gene for this disorder, inherited as autosomal recessive pattern. *SLC19A3* belongs to the *SLC19* (solute carrier family 19) gene family (comprising *SLC19A1, SLC19A2*, and *SLC19A3*), and encodes a 496 amino acid human thiamine transporter (hTHTR2), a second thiamine transporter. The protein is formed by 12 transmembrane domains flanked by cytoplasmic N- and C- terminal regions (Ganapathy et al., 2004). So far, over a hundred patients have been reported, mainly from Saudi Arab regions (Alfadhel and Tabarki, 2018), whereas rare Chinese cases were reported. In this study, we described the largest cohort of Chinese population with *SLC19A3* gene defect and reviewed all reported cases to elucidated its phenotypic and genetic spectrum.

## Methods

### Patients and Clinical Information

A total of 18 individuals with *SLC19A3* mutations were recruited, of whom 4 cases had been reported previously (Li et al., 2020). The study protocol was approved by the Ethic Committee of all hospitals involved. Informed consent of routine and investigative studies was obtained from parents of all studied patients and families. We also obtained consent from the parents of families for genetic studies. Clinical data, including age of onset, clinical manifestations, MRI findings, laboratory examination, family history, treatment and outcomes were collected.

### Molecular Analysis

Genomic DNA was extracted from the peripheral blood leukocytes of patients and their parents according to a standard protocol. A minimum of 3 ug DNA was used to make the indexed Illumina libraries according to the manufacturer’s protocol. The amplified DNA library was captured using a mitochondria genome and nuclear gene enrichment kit (MitoExome kit, MyGenostics, Beijing, China). The enrichment libraries were sequenced on Illumina HiSeq 2000 platform with 150-bp paired-end reads.

Raw reads were aligned to UCSC hg19 and the mitochondria genome (NC_012920) with BWA software. Aligned reads were processed with SAM tools and Picard following the best practice guidelines of the Genome Analysis Tool kit (GATK). Single-nucleotide variants and small insertion-deletions were detected with the GATK Haplotype Caller. Variants were annotated using ANNOVAR^1^. Common sites with population allele frequency above 5% according to dbSNP 138, 1000 Genome Project, esp6500si, and ExAC databases were excluded. Pathogenicity of variants was interpreted according to the ACMG Standards and Guidelines of 2015. To confirm the mutations identified by WES, fragments covering the mutation site in *SLC19A3* gene were amplified by PCR followed by Sanger sequencing.

To analyze the large fragment amplification or deletion in the mitochondria genome, the sequencing depth of each position on the mitochondria genome was plotted along the base position. The amplification or deletion was determined when an abnormal peak or dent was observed.

### Literature Review

A systematic literature review on *SLC19A3* gene defect was performed since 1998 on Pubmed, using “biotin responsive basal ganglia disease,” “biotin-thiamine responsive basal ganglia disease,” “thiamine transporter-2 deficiency,” or “*SLC19A3*” as search terms. Cases with adequate clinical documents, biochemical, radiographic and molecular results were rolled into the research. Clinical and genetic information was summarized in detail.

### Statistical Analysis

Statistical analyses were performed using IBM SPSS Statistics 22 software (IBM Corp., Armonk, NY). The quantitative variables were reported either in terms of the normal distribution mean, standard error of the mean, and the range. The Mann-Whitney *U*-test was applied to evaluate differences in numerical variables between groups. The chi-square test and Fisher’s exact test were used to test the association between categorical variables.

## Results

### Clinical and Genetic Profile of Chinese Cohort

A total of 18 unrelated patients were included, including 11 females and 7 males, of whom 4 patients have been described previously (Li et al., 2020). None of them had positive consanguinity.

The average age of disease onset was 0.91 ± 0.27 years (1 month–3.41 years). Fourteen (77.8%) patients initially presented with subacute encephalopathy (emotional change or altered level of consciousness), three patient (P129, P139, and P143) initiated as recurrent dyskinesia with spontaneous remission, and one patient (P142) only had global developmental delay since birth without any acute/subacute events attacked. Seizures occurred in eight patients (44.4%), including general tonic-clonic seizures (GTCS) or spasms. In our study, eight patients (44.4%) were diagnosed with BTBGD, nine patients (50%) presented with infantile Leigh-like syndrome or infantile spasms, and one patient (5.6%) was diagnosed with non-specific developmental delay.

The biochemical analysis showed high serum lactate levels in 56.3% (9/16) of patients. Cerebrospinal fluid (CSF) lactate was slightly elevated in one of seven patients. The routine indicators, including ammonia, creatine phosphokinase, metabolic profile, were unremarkable in all patients. Muscle biopsy was performed in one patient, which was unremarkable. Brain MRI was performed in 16 patients during acute phases, and in eight patients at post-acute stage.

Sixteen patients accepted vitamin supplementation. Time frame from onset to therapy ranged from several days to 22 months (mean = 4.57 ± 1.73 months). One patient (P10) was treated with biotin alone and eventually died. One patient (P11) recovered after thiamine supplementation. Fourteen patients were treated with combination of biotin (1–10 mg/kg.d) and thiamine (10–40 mg/kg.d). Five of them fourteen were symptoms-free or left with mild deficit which had no effect on daily life, seven with moderate to severe sequela and two deceased.

Four patients (22.2%) were detected with homozygous *SLC19A3* mutations and 14 (77.8%) with compound heterozygous variants. A total of 24 *SLC19A3* mutations were identified, including 16 (69.6%) missense mutations, 5 truncating mutations (nonsense or frameshift mutations), 2 splicing site mutations and a gross deletion of the whole *SLC19A3* gene. All the *SLC19A3* mutations were inherited from heterozygous parents. Only one mutation (c.980-14A > G) has been described in foreign cohort, and the rest 23 mutations were merely identified in Chinese patients.

The information about clinical presentation, ancillary testing, neuroimaging, treatment and outcome, as well as molecular results were summarized together in Supplementary Table 1 and compared with previous reports in the “Literature Review” section.

### Literature Review

#### Demography

**FIGURE 1:**
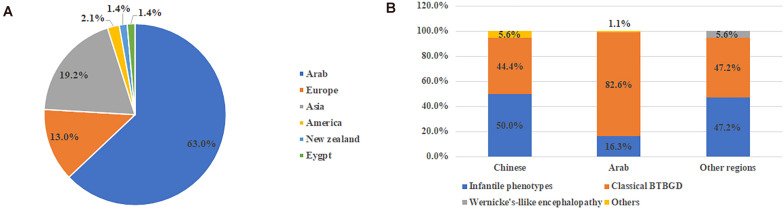
The ethnic origin of all published cases with *SLC19A3* gene defect **(A)** and category of clinical features in different ethnic background **(B)**. Other included non-specific developmental delay and asymptomatic.

Including our study, a total of 159 patients were diagnosed with THMD2 in 40 reports (Ozand et al., 1998; Adhisivam et al., 2007; El-Hajj et al., 2008; Bindu et al., 2009; Kono et al., 2009; Debs et al., 2010; Yamada et al., 2010; Serrano et al., 2012; Alfadhel et al., 2013; Fassone et al., 2013; Gerards et al., 2013; Kevelam et al., 2013; Perez-Duenas et al., 2013; Tabarki et al., 2013a, b, 2015; Distelmaier et al., 2014; Haack et al., 2014; Kassem et al., 2014; Ortigoza-Escobar et al., 2014, 2016, 2017; Schanzer et al., 2014; Sremba et al., 2014; Kohrogi et al., 2015; Aljabri et al., 2016; Bin Saeedan and Dogar, 2016; Bubshait et al., 2016; Flones et al., 2016; Schwarting et al., 2016; Ygberg et al., 2016; Alfadhel, 2017; Algahtani et al., 2017; Eichler et al., 2017; Pronicki et al., 2017; Whitford et al., 2017; Gowda et al., 2018; Tonduti et al., 2018; Savasta et al., 2019; Li et al., 2020). Thirteen patients were excluded, including 9 patients without genetic results and 4 patients without clinical information. Finally, 146 patients were used for analysis of clinical characteristics, including 67 females and 79 males. Over half (92, 63%) were from Arab, followed by Asia (28, 19.2%) and Europe (19, 13%) (Figure 1). Chinese patients accounted for 64.3% (18/28) of the patients with Asian background. In 58.1% (68/117) of patients, parents were consanguineous.

#### Clinical Categories

The age of onset ranged from birth to decades of years (mean = 3.66 ± 0.34 years), while the age of diagnosis was at mean of 7.21 ± 1.40 years, 4.12 ± 1.20 years after onset. Diagnosis was made after death in 21.9% (32/146) of patients. Pre-existing trigger factors were found in 44.8% (56/125) of patients, including infections, trauma, vaccination, profuse exercise, etc.

Of all 146 cases, 101 (69.2%) were diagnosed with classical BTBGD, 41 (28.1%) with more severe phenotypes, including infantile BTBGD, infantile Leigh-like syndrome, infantile spasms or neonatal lactic acidosis, which were referred as infantile phenotypes in the following paragraphs, and two (1.4%) with Wernicke’s-like encephalopathy. In addition, one patient was asymptomatic and one patient presented with non-specific developmental delay. Here, the clinical phenotype of each patient was defined according to the original literature.

From the view of ethnic background, patients of classical BTBGD accounted for the majority (82.6%, 76/92) in Arab cohort. In Chinese cohort and other regions, the proportion of BTBGD or infantile phenotypes accounted for about half, respectively (Figure 1B). Wernicke’-like encephalopathy was diagnosed in two patients from Japan. Here, we summarized the clinical information of each phenotype, separately.

#### Clinical Characteristics

**FIGURE 2:**
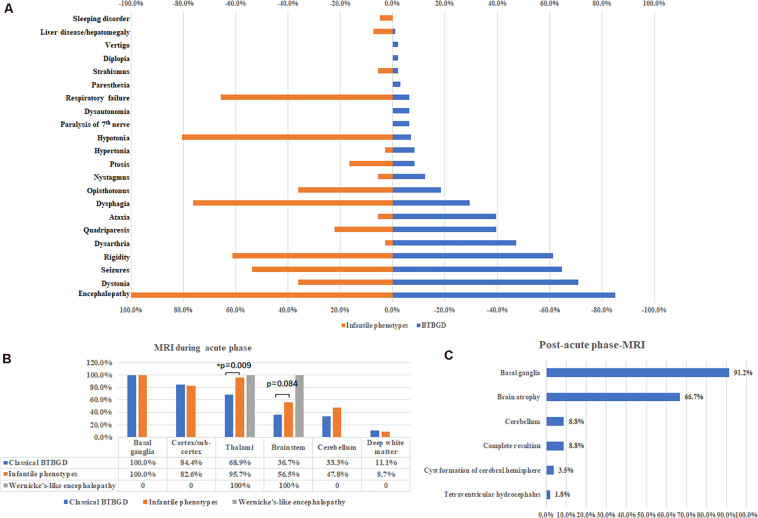
Major clinical features **(A)** and neuroimaging results during acute phases **(B)** and post-acute phases **(C)**.

1. **Classical childhood BTBGD:** A total of 101 patients were diagnosed with classical childhood BTBGD, including 51 females and 50 males. The age of onset of signs and symptoms ranged from birth to 12 years old (mean: 4.73 ± 0.35 years). Most patients presented with episodes of subacute encephalopathy with confusion, emotional alteration or progressive coma. Seizures occurred in over half of cases (myoclonic jerks, epileptic spasms, focal, and generalized seizure, epilesia partialis continua and status epilepticus). Other common symptoms included dystonia, rigidity of limbs, dysarthria, quadriparesis, ataxia, dysphagia, episodic opisthotonos. A small subset of patients presented with dysautonomia, abnormal breathing, or acroparesthesia. Rhabdomyolysis, liver disease and jaundice, and autistic behavior, depression was also described in single patients.
2. **Infantile phenotypes:** A total of 41 patients were classified into this group, including 15 females and 26 males. The symptoms started at 0.25 ± 0.04 years (18 days–13 months). All patients presented with encephalopathy with irritability, crying and screaming, or lethargy. Unlike classical BTBGD, hypotonia, dysphagia and respiratory failure was much more common. Other symptoms were similar to that of classical childhood BTBGD (Figure 2A).
3. **-like encephalopathy:** Only two Japanese patients has been described with Wernicke’s-like encephalopathy, who presented in their second decade of life with status epilepticus, diplopia, nystagmus, ptosis, ophthalmoplegia and ataxia.

#### Lab Examination and Neuroimaging

Blood lactate was elevated in 23.9% (26/109) of patients. Patients with hyperlactacidemia had much earlier onset of age (0.38 ± 0.10 years vs. 4.33 ± 0.39 years, *p* < 0.001, Mann Whitney *U*-test), of whom 92.3% (24/26) presented with infantile Leigh-like syndrome or infantile spasms, and 69.2% (18/26) deceased. A few patients showed increased cerebrospinal fluid (CSF) lactate (8 of 96 patients). Except one improved after therapy, almost all of them showed poor prognosis (5 patients deceased and 2 patients with severe neurological deficit). Other laboratory examinations including organic acid profiles, routine test was unremarkable.

Brain MRI was performed during acute crisis in 114 patients. Except two patients with Wernicke’s like encephalopathy, all cases showed symmetrically distributed brain lesions in basal ganglia. Other common involved regions included cortex and subcortical white matters, thalami, brain stem and cerebellum. Deep white matter was affected in some cases (Figure 2B). A small subset of cases showed thin corpus callosum, and abnormal signal at spinal cord. Compared with classical BTBGD, thalami were more frequently involved in patients with infantile phenotypes (62/90 vs. 22/23, Fisher exact test, *p* = 0.009). Although not significantly, the deceased patients seemed to have more frequent involvement of the brainstem than survivals (60%, 12/20 vs. 38.3%, 36 of 94, respectively). Brain MRI was performed at post-acute phase in 57 patients. Almost all patients demonstrated permanent anomalies of bilateral basal ganglia, except five patients showed complete resolution of lesions during acute phase. Global brain atrophy was seen in 66.7% of patients (Figure 2C).

Oxidative phosphorylation (OXPHOS) activity of muscle or skin fibroblast was available in 26 patients. Except five patients showed reduced activity of one or more complexes, and two showed reduced ATP synthesis, all the rest had no disturbance of respiratory chain complexes. All patients with anomalies of OXPHOS activity presented with infantile BTBGD or infantile Leigh-like syndrome, and six of seven deceased eventually (Gerards et al., 2013; Kevelam et al., 2013; Haack et al., 2014; Ygberg et al., 2016; Pronicki et al., 2017).

#### Treatment and Outcome

A total of 114 patients received vitamin supplementation, including 11 with biotin alone, 19 with thiamine alone and 84 with combination of two. The time from onset to therapy initiation varied from days to decades of years. Among them, 70 patients (61.4%) were symptoms-free or with mild deficit which had no effect on daily life, 33 patients (28.9%) had clinical improvement but with neurological sequela left of different degree, and 11 patients (9.6%) did not improve at all and deceased eventually.

**FIGURE 3:**
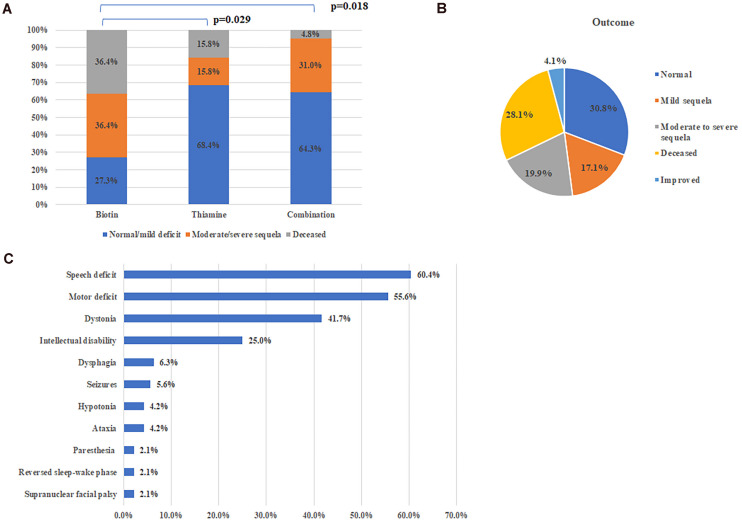
Treatment and outcome of all patients. **(A)** Therapeutic effects of different therapy. **(B)** Outcomes of all reported cases with SLC19A3 gene defect. **(C)** Major sequela in survivals.

Among patients who received treatment within 1 month after disease onset, the proportion (73.3%, 33/45) of patients with good recovery (symptoms-free or merely with mild deficit) was significantly higher than that (35.0%, 14/40, Chi-square test, *p* = 0.001) of patients with delayed therapy (There is no significant difference on the age of onset between the two groups, 4.3 ± 0.65 years vs. 3.2 ± 0.53 years, *p* = 0.257, Mann Whitney *U*-test). There is no significant difference on the ratio of patients with good recovery between groups of thiamine alone or combination with biotin (68.4%, 13/19 vs. 64.3%, 54/84), but both significantly higher than that (*p* = 27.3%, 3/11) with biotin alone (Figures 3A, Fisher’s exact test, *p* = 0.029 and *p* = 0.018, respectively).

On the whole, 104 patients (71.2%) were alive at the time of report, including 69 patients with satisfactory outcome (completely normal or with mild deficit), 29 patients with neurological sequela affecting daily life and specific situation were not available in 6 patients (Figure 3B). Common neurological sequela included speech deficit, motor deficit (from mild handicap to quadriplegia), dystonia and intellectual disability (Figure 3C). Compared with patients presented with early onset crisis, the survival rate was significantly higher in patients with classical BTBGD (88.1%, 89/101 vs. 26.8%, 11/41, Chi-square test, *p* < 0.0001). Almost all survivals with infantile phenotypes were left with severe sequela.

Forty-two patients (28.1%) died at 5.37 ± 1.11 years (2 months–26 years), of whom 71.4% (30/42) presented with infantile Leigh-like syndrome/infantile spasms/neonatal lactic acidosis/infantile BTBGD. The mean deceased age, for classical form and infantile form, was 8.89 ± 2.54 years (14 months–26 years) and 3.46 ± 1.04 years (2 months–20 years), which is 6.33 ± 2.51 years (1 months–22 years) and 3.37 ± 1.05 years (few days–20 years) after disease onset, respectively. The time from onset to death has no significant difference between two groups (Man-Whitney *U*-test, *p* = 0.207). The direct cause of death was clearly described in 28 patients, of which 92.9% (26/28) were respiratory complications (abnormal breathing pattern, respiratory tract infection). Other factors consisted of septicemia, cardiac arrest, status epilepticus. Of all 42 deceased cases, 30 (71.4%) died before vitamin therapy initiation.

#### Genetic Profile

**FIGURE 4:**
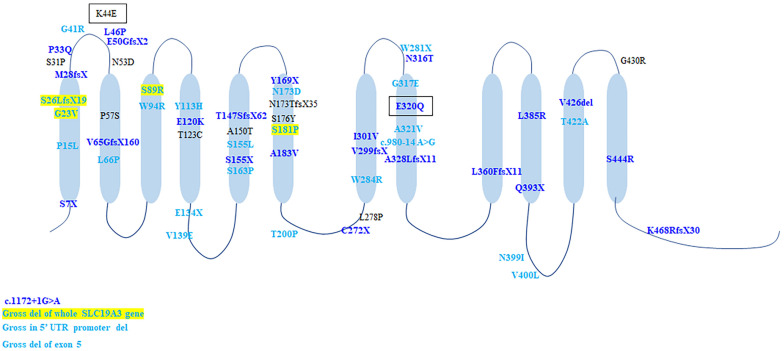
The schematic of *SLC19A3* protein and the location of all identified mutations. Mutations in light blue were discovered in patients with classical BTBGD. Mutations in dark blue were found in patients with infantile phenotypes. Mutations in yellow box were found in both classical and infantile phenotypes. Mutations in black box were found in Wernicke’s-like encephalopathy.

A total of 60 gene mutations were found in *SLC19A3* gene, including 37 (61.7%) missense mutations and 17 (28.3%) truncating mutations, 2 splicing mutations, two gross deletions affecting the promoter and two gross deletion of exons and the whole gene. In Chinese cohort, 24 mutations were identified, of which 23 mutations were novel. All mutations scattered along the *SLC19A3* gene (Figure 4). There were 25 patients with two truncated mutations. Except four patients with (c.980-14A > G, p.S26LfsX19), who presented with mild phenotype and normal outcome, all the rest patients (21/25, 84%) presented with infantile phenotypes. Compared with biallelic missense mutations, biallelic truncated mutations resulted in earlier onset of disease (1.38 ± 0.62 years vs. 4.02 ± 0.35 years, Mann-Whitney *U*-test, *p* < 0.001) and worse outcome (survival rate: 92/110 vs. 7/25, Chi-square test, *p* < 0.001).

## Discussion

Basing on next generation sequencing collected in the Exome Aggregation Consortium, Ferreira estimated that *SLC19A3* gene deficiency has a high prevalence of about 1 of 215,000 live births (Ferreira et al., 2017). There is not yet epidemiological investigation or calculation of sequencing data in Chinese population. Given basis of the huge population in China, it is likely that the majority remains underdiagnosed. Here we described the largest Chinese cohort of *SLC19A3* gene deficiency, which contributed over half of the cases with Asian background.

Including our study, more than 100 cases has been described. Although the symptoms onset at average of 3.66 years old, the diagnosis was made approximately 4 years later on average, and even more than 1/5 of the cases were not diagnosed before their death, which indicated that clinicians had insufficient understanding of this disease. As a treatable metabolic disease, early recognition and intervention are very important for the prognosis. Here we summarized all reported cases, to further elucidate its clinical characteristics, especially the prognostic factors.

*SLC19A3* gene deficiency is associated with a continuum of clinical syndrome with variable outcomes (Alfadhel and Tabarki, 2018). Through review of the literature, patients of classical form accounted for 69.2%. But the proportion in Arab cohort (82.6%) is much higher than that of Chinese (44.4%). Meanwhile, the onset age of our cohort was earlier than that of Arabians. This might be explained by the hot spot mutation T422A in Arab population, which resulted in relatively uniform and mild phenotypes, whereas the mutations are much more scattered in Chinese cohort.

The clinical course of classical BTBGD was variable, with overwhelming majority of patients had episodes of encephalopathy. Our cases demonstrated a broad range of onset age, clinical presentation and outcome. Interestingly, several patients initiated as recurrent dyskinesia with spontaneous remission. The literature review showed that a minority of patients had an insidious onset of symptoms, such as ptosis (Fassone et al., 2013), hyperactivity and attention deficit, episodic stiffness of limbs, which remised spontaneously (Ozand et al., 1998). Some patients initially presented with seizures or pure ophthalmoplegia (Tabarki et al., 2013b). The clinical pictures in patients with infantile phenotypes were almost the same, all manifesting an acute devastating course during early months of life, with feeding difficulties, inconsolable crying or lethargy, loss of milestones. Respiratory failure and hypotonia were more common in patients of this group (Yamada et al., 2010; Perez-Duenas et al., 2013; Gerards et al., 2013; Kevelam et al., 2013; Sremba et al., 2014; Ygberg et al., 2016; Alfadhel, 2017; Algahtani et al., 2017; Pronicki et al., 2017; Savasta et al., 2019). Wernicke’s-like encephalopathy was only described in two Japanese patients (Kono et al., 2009).

Lactic acidosis was common in patients with early encephalopathies and indicated poor outcome (Perez-Duenas et al., 2013; Gerards et al., 2013; Kevelam et al., 2013; Sremba et al., 2014; Ygberg et al., 2016; Alfadhel, 2017; Tonduti et al., 2018; Savasta et al., 2019). Brain MRI is abnormal in all symptomatic cases. Bilateral basal ganglia were involved in all patients except two with Wernicke’s-like encephalopathy. The less frequently involved regions were cortical and subcortical regions, followed by thalami, brain stem/midbrain, cerebellum and deep white matters. Brainstem was more frequently involved in patients with poor prognosis. These auxiliary results are highly suggestive of mitochondrial disorders. A number of patients were initially diagnosed with mitochondrial disease, and received cocktail therapy containing biotin, which might be partially beneficial. However, normal skin or muscle biopsy performed for respiratory chain enzymes activity in most cases made the diagnosis of mitochondrial disorders less likely. Therefore, muscle or skin biopsy was recommended for distinguish diagnosis. Abnormal skin/muscle OXPHOS activities in a small part of patients usually led to infantile phenotypes and poor prognosis (Gerards et al., 2013; Kevelam et al., 2013; Sremba et al., 2014; Ygberg et al., 2016; Pronicki et al., 2017).

*SLC19A3* gene deficiency as a treatable condition, timing of intervention is critical. The literature review showed that approximately three quarters of patients had good recovery and high life quality if treated in time. However, delayed therapy led to irreversible brain damage and catastrophic outcome. Almost all deceased cases were undiagnosed and untreated.

In terms of treatment, it is complex and ambiguous about the dosage of biotin and thiamine in the treatment of this disease. Actually, in original reports, thiamine was not effective. Ozand et al. (1998) reported that 5/10 patients were normal with high doses (5–10 mg/kg.d) of biotin alone over a long period of follow-up. Some patients were reported improving significantly after the treatment of biotin in lower dosage (5–30 mg/d) (El-Hajj et al., 2008). Debs et al. (2010) reported two siblings with the same mutation, of whom one responded well to biotin alone, while the other one did not improve until the addition of thiamine. From our study and literature review, although beneficial to someone, the therapeutic effect of biotin alone is much poorer than thiamine, while the combination of biotin and thiamine is not superior to thiamine alone, which is coincident with the study of Tabarki et al. (2015). From a genetic point of view, *SLC19A3* encodes the human thiamine transporter, while biotin is not its substrate (Subramanian et al., 2006). However, the mechanism underlying which a part of patients improved dramatically on biotin remains unclear. Perhaps the functionality of biotin and thiamine transporters is closely coupled. However, no direct evidence is yet available to support such a linkage. But from the clinical view, it is recommended that the treatment regimen contain both thiamine (10–40 mg/kg.d) and biotin (1–10 mg/kg.d).

On the other hand, early onset of disease usually indicated poor prognosis. The survival rate was 19.4% (7/36) of patients with early onset phenotype, significantly lower than that (87.7%, 93/106; chi-square test, *p* < 0.001) of patients with classical form. Over half of them passed away within 1 year since onset. Rare cases responded well to therapy, and even early therapy cannot stop the deterioration in most of them. Although not significant, the lifespan seems shorter than patients with classical phenotype (3.37 ± 1.05 years vs. 6.33 ± 2.51 years).

Genotypic correlation remains unclear, but biallelic truncated mutations usually results in early onset and poor prognosis. The most prevalent genetic alteration is T422A, identified in homozygosity in over half (52.1%, 76/146) of patients, all with Arab ethnic backgrounds. All patients presented with classical BTBGD. G23V was found in nine patients with different backgrounds (Arab, Canada, Poland, Italy, India, New Zealand), homozygous in six patients and compound heterozygous in three patients (Ozand et al., 1998; Serrano et al., 2012; Kevelam et al., 2013; Pronicki et al., 2017; Whitford et al., 2017; Gowda et al., 2018; Tonduti et al., 2018). The age of onset was relative earlier (mean = 9.94 ± 2.25 months, 1 month–2 years). The clinical course and outcome were variable. Even in the same family, the patients showed distinctly different phenotype and responses to vitamin supplementation (Gowda et al., 2018). S7X occurred exclusively in patients (*n* = 9) of Morocco presented with early infantile Leigh-like encephalopathy, who all died soon after disease onset (Gerards et al., 2013). Homozygous E320Q was described in Japanese patients with early onset encephalopathy and lead to early death (Yamada et al., 2010), whereas compound heterozygous E320Q combined with K44E lead to adult-onset Wernicke’s-like encephalopathy and benign clinical outcome (Kono et al., 2009). The splicing mutation, c.980-14A > G, was found in 5 patients of Europe origin (*n* = 5), in compound heterozygous condition with a frame shift mutation (S26LfsX19/L360PfsX38) (Debs et al., 2010; Serrano et al., 2012; Ortigoza-Escobar et al., 2014), as well as one Chinese patient, combined with a missense mutation (S89R). All except one presented with classical form and had good response to biotin and thiamine. Nevertheless, the mutation c.980-14A > G allele showed the total exclusion of exon 4 and caused a truncated protein (G327NfsX8), which lead to severe impairment of hTHTR2 function (Ortigoza-Escobar et al., 2014). It might be hard to explain the contrast between the biallelic truncation and the relative milder phenotype. Hence, it is probable that a combination of yet unknown genetic and environmental factors may be jointly involved in the genetic interaction network that sustains the disease phenotype. Missense mutation A321V was identified in 4 Chinese patients, of whom one was homozygote and three were compound heterozygous with another missense mutation. All four of them presented with classical BTBGD and had satisfactory outcomes. A splicing mutation c.1172 + 1G > A was identified in two patients with early infantile form, both in compound heterozygous condition. It can be seen that the genotypic analysis is hard to implement. Accumulation of genetic analysis and clinical courses of patients with *SLC19A3* gene defect, as well as laboratory studies employing cellular or animal models will be needed to better characterize the correlation between molecular structure and clinical manifestation.

In conclusion, this study described the largest Chinese cohort of THMD2, which expand the genetic and clinical spectrum of the disorder. Literature review revealed that elevated lactate in blood and CSF, as well as abnormal OXPHOS activities of muscle or skin usually correlated with infantile phenotypes, which indicated poor outcome. Brainstem involvement on MRI was more common in deceased cases. Thalami was more frequently involved in infantile phenotypes. As a treatable disorder, early diagnosis and intervention is critical to improve the prognosis. Thiamine supplementation is indispensable in the treatment of THMD2, whereas combination of biotin and thiamine is not superior to thiamine alone. Biallelic truncated mutations usually led to more severe phenotype, but deeper genotypic-phenotypic correlation needs further investigation.

## Data Availability Statement

The data that support the findings of this study are available from the corresponding author upon reasonable request.

## Author Contributions

JPW, JLW, XH, and ZL collected the clinical data of all patients. YM, GC, HZ, DS, RX, YL, YZ, and YW acquired samples from the patients. ZL also made contribution in genetic analysis. GC, DS, RX, YL, YZ, and XB provided help in clinical evaluation of the patients. JPW made routine follow-up of the patients and drew up the manuscript. FF and QC designed the study and made contribution in English editing of the manuscript. All authors have read and approved the manuscript and agreed with its publication.

## Conflict of Interest

The authors declare that the research was conducted in the absence of any commercial or financial relationships that could be construed as a potential conflict of interest.

## Footnotes

1 http://annovar.openbioinformatics.org/en/latest/
